# Respiratory and Neurological Disease across Different Ethnic Groups Is Influenced by the Microbiome

**DOI:** 10.3390/microorganisms9091965

**Published:** 2021-09-16

**Authors:** Odiase Peace, Kartik Rachakonda, Miller Kress, Fernando Villalta, Girish Rachakonda

**Affiliations:** 1 Department of Microbiology, Immunology and Physiology, Meharry Medical College, Nashville, TN 37208, USA; opeace20@email.mmc.edu (O.P.); fvillalta@mmc.edu (F.V.); 2 School of Arts and Science, Vanderbilt University, Nashville, TN 37212, USA; kartikracha7@gmail.com; 3 División of Molecular Diagnosticas, Phase2Labs, Nashville, TN 37217, USA; miller.kress.22@harpethhall.org

**Keywords:** nasal microbiome, allergic rhinitis, microbial dysbiosis, asthma, immune response, health disparities, *Moraxella*, *Staphyloccus aureus*, neurological disease, race or ethnicity

## Abstract

Acute and chronic upper respiratory illnesses such as asthma, and allergic rhinitis (AR) have been linked to the presence of microorganisms in the nose. Microorganisms can exist in symbiotic or commensal relationships with the human body. However, in certain cases, opportunistic pathogens can take over, leading to altered states (dysbiosis) and causing disease. Thus, the microflora present in a host can be useful to reflect health status. The human body contains 10 trillion to 100 trillion microorganisms. Of these populations, certain pathogens have been identified to promote or undermine wellbeing. Therefore, knowledge of the microbiome is potentially helpful as a diagnostic tool for many diseases. Variations have been recognized in the types of microbes that inhabit various populations based on geography, diet, and lifestyle choices and various microbiota have been shown to modulate immune responses in allergic disease. Interestingly, the diseases affected by these changes are prevalent in certain racial or ethnic populations. These prevalent microbiome variations in these groups suggest that the presence of these microorganisms may be significantly associated with health disparities. We review current research in the search for correlations between ethnic diversity, microbiome communities in the nasal cavity and health outcomes in neurological and respiratory functions.

## 1. Introduction

Complex relationships exist between health and biology, genetics and individual behavior. Host lifestyle preferences and hygiene, as well as access to health information and health service, socioeconomic status, environment, discrimination, racism, literacy levels and legislative policies, can impact the health status of an individual or a population group. Disparate geographical surroundings, including physical, chemical and psychosocial factors, impact health choices leading to changes in health outcomes by influencing the severity of disease across racial groups. These aforementioned factors, including the host’s immune system, have also been implicated in observed human microbiome shifts [1]. Health disparities are differences in the health status of groups of people based on factors such as race, ethnicity, age, sex, socio-economic status, geographic location, mental health, disability, citizenship status or other characteristic linked historically to exclusion or discrimination [2]. There are large racial and ethnic differences in health in the United States, and certain health conditions or diseases tend to be prevalent within specific population groups [3]. Despite this, not much is known about agents that spark microbiome alterations across many disease states and not a lot of research has been undertaken to fully understand how racial and ethnic health disparities are linked to variations in the microbiome. This is important as racial and ethnic health differences permeate every social economic stratum. Diversity in diet, and other habits that shape health which are impacted by cultural, ancestral and social frameworks may determine microbiome differences and cause health disparities. Previous research studies have categorized environmental factors that influence health disparities and even certain microbiome variations in particular ethnic groups. For example, single gene mutations in sickle cell disease (SCD) are most common in Blacks and African Americans (AAs) owing to ancestral linkage to genetic adaptation to environmental factors such as malaria-causing anopheles’ mosquitos in Africa [4]. Similarly, SCD is also observed among Indian and Middle Eastern Arab populations owing to the endemicity of malaria [5,6]. SCD patients, when compared with carriers of the sickle cell trait, showed differences in microbiota composition in the top 15 genera that accounted for 84% of the taxonomic abundance across all samples. *Pseudobutyrivibrio* (*p* = 0.05), *Faecalibacterium* (*p* = 0.11), *Subdoligranulum* (*p* = 0.16) (all phylum Firmicutes), *Prevotella 9* (*p* = 0.07), and *Alistipes* (*p* = 0.04) (both phylum Bacteroidetes) were lower in abundance, while *Escherichia*-*Shigella* (*p* = 0.11) (phylum Proteobacteria) was higher in abundance in SCD [7]. Vaginal characterization amongst women of different ethnic backgrounds revealed variations in vaginal pH and the microbiota in European women are more likely than AA women to be *Lactobacillus*-dominated, which appeared to maintain vaginal health [8]. These highlighted studies reveal interesting features of the microbiome in various ethnic populations and show how understanding social and environmental factors can influence our understanding of disease. Very little is known about the impact of nasal microbiota on immune responses in the host. Yet, variations in nasal microbiota are observed among ethnic groups with associated disparities in the prevalence of diseases. For example, rheumatoid arthritis, an autoimmune disorder, has been associated with changes in the oral and gut microbiomes which influence the loss of tolerance against self-antigens and impact the inflammatory events that aid the damage of joints. Interestingly, rheumatoid arthritis occurs in varying levels among various ethnic groups in the United States. Significant differences of mean disease activity level (*p* < 0.001) were observed across racial and ethnic groups and these differences persisted (*p* < 0.046) even though improvements in disease activity were observed in all groups over a 5-year period. Remission rates also remained significantly different across racial/ethnic groups across all models ranging from 22.7 (95% Confidence Interval (CI) 19.5–25.8) in AAs to 27.4 (95% CI 24.9–29.8) in CAs [9]. Since the microbiome has immunomodulatory functions, an imbalance or dysbiosis in microbial community structure could be the driving force behind diseases [10]. Thus, we aim to analyze the information from the current/existing literature on nasal biome variations and investigate its role in health disparities by considering its impacts on the physiological and biological processes. Furthermore, we aim to evaluate the social and physical environmental factors that influence the genesis of such microbiome variations, especially within ethnic groups. Revealing the influence that the microbiome has on the existence of health disparities could shed light on the increased prevalence of certain diseases in some populations and improve our comprehension of why certain ethnic groups have greater disease risk and fatality compared to others.

## 2. Microbiome Populations in the Nose and Impacts of Dysbiosis in Disease States

**Figure 1:**
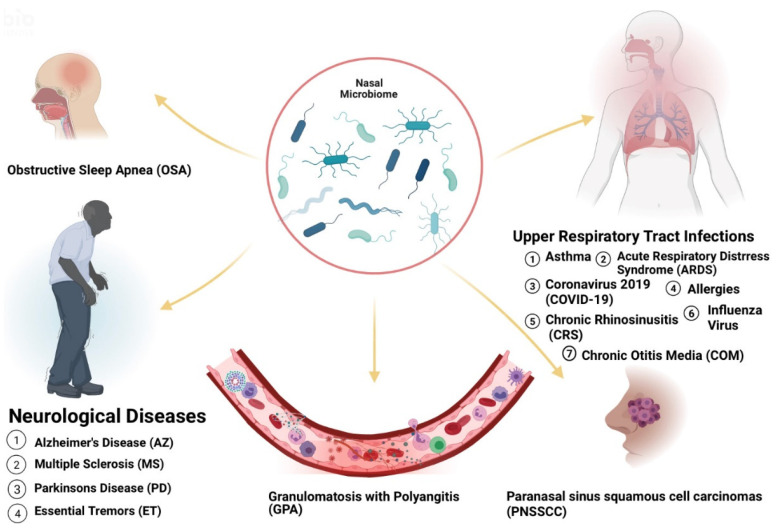
Several diseases are linked to dysbiosis in nasal microbiota.

The human body hosts a variety of microorganisms that reside in helpful, harmful, or otherwise commensal relationships within the body. The human microbiome refers specifically to the complex community of over 100 trillion microorganisms, living in human microhabitats [11]. Due to microbial niche specificity, the composition of microbes within a microenvironment differs based on the location on the human body, such as the gastrointestinal tract, skin and airways. Analysis of bacterial populations in the anterior nares from metagenomic samples revealed the presence of four dominant bacterial communities: *Moraxella*, *Staphylococcus*, *Propionibacterium* and *Corynebacterium* [12]. In Finland, five nasal microbiota profiles were observed in infants namely *Moraxella*, *Streptococcus*, *Dolosigranulum*, *Staphylococcus* and *Corynebacteriaceae* [13]. Other bacteria detected in the sinuses included *Firmicutes*, *Proteobacteria* and *Actinobacteria* phyla. On the species level, *Staphylococcus epidermidis*, *Propionibacterium acnes* and *Staphylococcus aureus* were the most prevalent. *Corynebacterium tuberculostearicum* was the most dominant of the *Corynebacterium* phyla. Opportunistic pathogens including *Streptococcus pneumoniae*, *Haemophilus influenzae*, *Moraxella catarrhalis*, *Stenotrophomonas maltophilia*, *Enterobacter species* were also found in healthy individuals [12]. Many studies report that the nasal microbiome of healthy humans is primarily composed of the phyla *Actinobacteria*, *Bacteroidetes*, *Firmicutes* and *Proteobacteria* with representatives of genera *Bifidobacterium*, *Corynebacterium*, *Staphylococcus*, *Streptococcus*, *Dolosigranulum* and *Moraxella* predominating. Significant alterations in the microbial community structure have been linked to the development and progression of disease [14] (Figure 1).

Nasal bacterial communities also show spatial variation, dependent on epithelium type. In adenoid and tonsillar tissue, *Haemophilus influenzae* primarily diffusely infiltrated the tissue, *Streptococcus* and *Bacteroides* were chiefly found in fissures, and *Fusobacteria, Pseudomonas* and *Burkholderia* were exclusively located within adherent bacterial layers and infiltrates [15]. In terms of the pathology of infection, microbial dominance plays a chief role (Table 1).

Interestingly, unique microbiota signatures exist within ethnic groups in the United States. Nasal microbiota has not yet been successfully characterized within ethnic groups in the United States, however, Mason et al., undertook a study using a machine learning classifier to distinguish between ethnicity of individuals based on their salivary samples. The classifier identified African Americans with a 100% sensitivity and 74% specificity and Caucasians with a 50% sensitivity and 91% specificity signifying that our microbiome may be genetically influenced and can act as an ethnic fingerprint [55]. Although nasal and oral microbiota have obvious discrepancies due to distinct environments for bacterial adhesion, survival, and growth [56], the differences observed are broadly mirrored disparities in each taxon’s signal abundance at each site. Since both the oropharynx and nostril collect drainage from shared sources (nasopharynx, sinuses, and nasal cavity), there is a major overlap in the taxa seen [57]. Mason et al. discovered that ethnicity was a crucial determinant of oral microbial colonization and that there exists a significant association between ethnicity and the composition of the oral microbiome among AAs, CAs, Chinese (CH) and LAs living within the United States. The abundances of four species level operational taxonomic units (OTUs) and several genus level-OTUs were significantly different between the ethnicities (* *p* < 0.05, ** *p* < 0.01, *** *p* < 0.001, **** *p* < 0.0001). Importantly, AAs demonstrated significantly lower bacterial diversity (*** *p* < 0.001, ANOVA) and equitability (*** *p* < 0.001, ANOVA) compared to the other ethnicities [55]. This indicates that AAs have fewer subgingival species and that a few of these species are numerically dominant members of the community when compared to the other ethnicities. Decreased diversity is considered an indicator of an unhealthy microbiome and has been linked to different chronic conditions such as decreased gut microbiota diversity in obesity and type 2 diabetes [58]. Thus, AAs may be more susceptible to respiratory illnesses in the United States.

### 2.1. Asthma

In children diagnosed with asthma, at least 95% hosted pathogenic genera *Moraxella*, *Staphylococcus*, *Streptococcus*, and *Haemophilus* in the nose. Different asthma phenotypes were significantly associated with variations in phyla (*Proteobacteria*, *Actinobacteria* and *Bacteroidetes*) abundances, community composition, structure, and co-occurrence of bacterial genera (*Moraxella*, *Corynebacterium*, *Dolosigranulum* and *Prevotella*) [59]. A predominant *Corynebacterium* population led to better asthma control compared to those with *Moraxella*. As exacerbation rates worsened, the microbiome population switched from *Corynebacterium* to a higher relative abundance of *Moraxella* [19]. In older (7.5 years old) children, the development of asthma was associated with an increase in *H.influenzae* colonization at 13 months of age. This association was not observed with *H.influenzae* colonization at 2 months of age [60]. The dominance of *Moraxella* during ages 2 to 13 months was also associated with a higher risk of developing childhood asthma [61]. In the United States, childhood asthma, affects one group of people differently than another group. Although childhood asthma is prevalent across all ethnic groups, the disease burden is disproportionately shared by Puerto Ricans (PRs) (19.2%) or African Americans (12.7%) compared to Caucasian Americans (CAs) (8%) or Mexican Americans (MAs) (6.4%) [20]. Ethnic disparities in asthma mortality rate are even greater with asthma mortality rates in children and adults in AAs being nearly eightfold and threefold higher, respectively, than in CAs [62]. The saliva of AA children with or without asthma was analyzed to compare bacterial diversity within the same demographic due to difficulty in obtaining nasal samples. Saliva samples from asthma cases were more enriched in pathogenic species compared to healthy controls. Differences between cases and controls were revealed in terms of diversity, as well as in relative abundance for *Streptococcus* genus (13.0% in cases vs. 18.3% in controls, *p* = 0.003) and *Veillonella* genus (11.1% in cases vs. 8.0% in controls, *p* = 0.002) [63]. Interestingly, no correlation with *Moraxella*, *Neisseria* or *Haemophilus* genera was found. The phylum in which these genera, Proteobacteria, were, however, included, and showed a trend to be more abundant in cases (27.7%) than in controls (25.4%) [63]. Risk factors include allergy or atopy, upper respiratory tract infection, snoring, acute otitis media, passive smoke exposure and low social status [21,22].

### 2.2. Acute Respiratory Illnesses

Influenza and other respiratory viral infections are the most common type of acute respiratory infection with great effects on host immune mechanisms. Acute respiratory disease (ARI) was shown to develop more frequently in children with early *Moraxella*-dominant profiles and less in *Corynebacteriaceae*-dominant profiles [13]. *Streptococcus* species and *Prevotella salivae* were additionally associated with the greater susceptibility to influenza B viral (IBV) infection and decreased susceptibility to influenza A viral (IAV) infection [35] ARIs such as Influenza induce antiviral immune responses that are associated with changes in microbial composition and function. These changes in the immune response also predispose patients to secondary bacterial infections, which are typically clinically more severe [64]. After adjusting for sex, age, race, disease severity, type of hospital and median household income for patient ZIP code, AAs had a greater odds ratio of in-hospital death for sepsis-related respiratory failure when compared with CAs (odds ratio [OR], 1.13; 95% CI, 1.11–1.14; *p* < 0.001), and LAs also had a greater odds ratio of in-hospital death when compared with CAs (OR, 1.17; 95% CI, 1.15–1.19; *p* < 0.001), and so did Asian and Pacific Islanders (OR, 1.15; 95% CI, 1.12–1.18; *p* < 0.001) and Native Americans (OR, 1.08; 95% CI, 1.00–1.15; *p* < 0.001) when compared with CAs (OR, 1.0) [22]. These variations may be related to complex interactions between an altered microbiome, virus-induced changes in immune response and growth of pathogenic bacteria as microbial diversity decreases [65]. Diabetes and Chronic respiratory disease also influence the occurrence of Influenza [37] Differences in Acute Respiratory Distress Syndrome (ARDS) incidence and associated mortality were also observed among different racial/ethnic affiliations. Among 96,350 patients studied, discrepancies were found among AAs and CAs for ARDS incidence (0.70% vs. 0.93%) and between LAs and CAs for ARDS-associated mortality (0.27% vs. 0.17%). There appeared to be a protective effect of AA race/ethnicity for ARDS incidence (OR, 0.73; 95% CI, 0.58–0.91) [38]. Risk factors for ARDS include abuse of alcohol and tobacco, malnutrition, and obesity [39].

Coronavirus disease 2019 (COVID-19), an infectious disease caused by binding of the viral spike (S) protein of the coronavirus to ACE2 receptors highly expressed in nasal goblet and ciliated cells, also correlated with microbial disparities in patients. In a study, *Deinococcus thermus* showed exclusive presentation only in controls when compared to COVID-19 patients admitted to intensive care unit (ICU), paucisymptomatic or affected by other coronaviruses. *Candidatus saccharibacteria* (formerly known as TM7) was strongly increased in negative controls and COVID-19 paucisymptomatic patients as compared to COVID-19 ICU patients. *Bifidobacterium* and *Clostridium* were completely depleted only in ICU COVID-19 patients and, *Salmonella*, *Scardovia*, *Serratia* and *Pectobacteriaceae* were observed only in ICU COVID-19 patients [26]. Likewise, there was a differential increase in species in patients with COVID-19 infection. *Peptoniphilus lacrimalis*, *Campylobacter hominis*, *Prevotella 9 copri* and *Anaerococcus* were more abundant in those with high viral load and COVID-19 infection whereas *Staphylococcus haemolyticus*, *Prevotella disiens* and *Corynebacterium* variants were more abundant in those with low viral load and without COVID-19 infection [27]. Ethnic disparities were also recorded in the United States. Using a large database containing 18,728,893 patients from 50 studies, AAs and AS ethnicities had a higher risk of COVID-19 infection compared to CAs individuals (pooled adjusted relative risk (RR) for AAs: 2.02, 95% CI 1.67–2.44; pooled adjusted RR for AS: 1.50, 95% CI 1.24–1.83) and sensitivity analyses examining peer-reviewed studies only (pooled adjusted RR for AA: 1.85, 95%CI: 1.46–2.35; pooled adjusted RR for AS: 1.51, 95% CI 1.22–1.88 [28]. Risk factors include hypertension, coronary artery disease, history of stroke, diabetes, obesity, severe obesity, chronic kidney disease, asthma and chronic obstructive pulmonary disease [29].

### 2.3. Rhinitis and Chronic Rhinosinusitis

Early childhood studies reveal that a decrease in bacterial diversity in infants over the period of 18 months, was associated with the development of rhinitis. Furthermore, symptoms associated with rhinitis were observed in infants with a depleted abundance of *Corynebacterium* [66]. Chronic rhinosinusitis (CRS) patients, however, showed an increased abundance of *Corynebacterium* [23]. Postoperatively patients with CRS who had better outcomes presented with greater bacterial diversity with higher relative abundances of *Actinobacteria* at the time of surgery [67]. LAs with CRS have greater disease severity and morbidity compared with CAs. Analysis of the nasal microbiota of CRS patients revealed that the nares of Latino patients were significantly less diverse compared to CAs (adjusted *p*-value = 0.03) and had a significantly higher relative abundance of *Burkholderia* genus compared to CAs (adjusted *p*-value = 0.04) [24]. Risk factors associated with CRS include asthma, gastroesophageal reflux disease (GERD), genetic polymorphisms, rheumatoid arthritis, migraine and cigarette smoking [25].

### 2.4. Otitis Media

Otitis media (OM) is a common pediatric diagnosis. Several risk factors have been associated with OM including lack of breast-feeding practices, daycare, mother multiparity and ethnic affiliation [34]. The association between OM diagnosis and race/ethnicity in 11,349 non-low-birthweight infants was measured. After adjustment for relevant confounders, AA (OR 0.74; 95% CI 0.61–0.89) and AS infants (OR 0.77; 95% CI 0.57–1.0) were less likely to be diagnosed with OM than CA infants [34].

Children with chronic otitis media with effusion (COME) had profiles that were *Corynebacterium*-dominated, *Streptococcus*-dominated or *Moraxella*-dominated when compared with healthy candidates. *Haemophilus influenzae*, *Streptococcus pneumoniae*, *Moraxella catarrhalis* and to a lesser extent *Staphylococcus aureus* were identified as principal composites involved in otitis. In contrast, higher abundances of *Haemophilus*, *Moraxella* and *Neisseria* in the nose have been found to be associated with acute OM (AOM) with lower abundances of *Staphylococcus*, *Corynebacterium*, *Propionibacterium*, *Streptococcus* (usually not *S. pneumoniae*) and *Lactococcus* [16]. There are several risk factors recorded for chronic OM (COM) such as the presence of allergy or atopy (OR, 1.36; 95% CI, 1.13–1.64; *p* = 0.001), an upper respiratory tract infection (OR, 6.59; 95% CI, 3.13–13.89; *p* < 0.00001), Snoring (OR, 1.96; 95% CI, 1.78–2.16; *p* < 0.00001), patient history of AOM (OR, 11.13; 95% CI, 1.06–116.44; *p* = 0.04), passive smoke exposure (OR, 1.39; 95% CI, 1.02–1.89 *p* = 0.04) and low social status (OR, 3.82; 95% CI, 1.11–13.15; *p* = 0.03) [18]. Chronic suppurative otitis media have also been shown to appear peculiar in West Africa [17].

### 2.5. Granulomatosis with Polyangiitis

Similarly, higher abundance of *S.aureus* was found in patients with active granulomatosis with polyangiitis (GPA) compared to healthy controls who had a higher abundance of *S.epidermidis* and *Propionibacterium acnes* (*p* = 0.04) [30]. Patients with active GPA (66.7%) had more *S.aureus* colonization compared with inactive GPA (34.1%) and nasal microbiota composition differed substantially between GPA patients and healthy controls (*p* = 0.039). Interestingly, the incidence was not significantly different in the AA/Minority Ethnic population from that in the CA population in the United Kingdom (adjusted odds ratio = 0.78, 95% CI: 0.53, 1.13, *p* = 0.13) [31]. Risk factors strongly associated with GPA included exposure to animals, especially horses (OR 3.08, 95% CI 1.34–7.08), a history of bronchiectasis up to 5 years before GPA diagnosis (OR 5.1; *p* < 0.0001), pulmonary fibrosis in the previous 3 years (OR 5.7; *p* = 0.01) and a previous diagnosis of an autoimmune disease or chronic renal impairment [32,33].

### 2.6. Atopic Dermatitis and Psoriasis

A *Moraxella*-dominant profile was also associated with increased severity in pediatric atopic dermatitis (AD). Correlations between nasal microbiota and skin microbiota were also found although the nose and skin harbor distinct microbial communities (*n* = 48 paired samples; *p* < 0.001) [49]. Persistent *S. aureus* colonization was also recorded in adult patients with AD with the same protein A gene type expressed both nasally and on the skin [49]. AD is a risk factor for colonization of nasal mucous membranes and the skin by methicillin-resistant *S. aureus* [68]. Higher overall rates of AD were found in Africa and Oceania compared to India and Northern and Eastern Europe. In the United States, AD prevalence was higher in AA (19.3%) compared with CA (16.1%) children. The immune phenotype of all ethnic groups was characterized by strong T_H_2 activation, however, important differences in immune polarization exist among the different ethnicities. AS patients with AD had stronger T_H_17/T_H_22 activation than AA and CA patients with AD, whereas AA patients had the highest serum IgE levels among all groups, while largely lacking T_H_1 and T_H_17 activation [50]. Risk factors associated with AD include viral and bacterial skin infections [69]. Interestingly, AD was also shown to be a risk factor for developing attention-deficit hyperactivity disorder (hazard ratio (HR) = 2.92, 95% CI = 2.48–3.45) or autistic spectrum disorder in children (HR = 8.90, 95% CI = 4.98–15.92) when aged 3 years or older [70].

Compared to healthy skin bacterial communities, psoriatic lesion and non-lesion skin also showed unique microbial signatures, with higher diversity and more heterogeneity compared to healthy skin bacterial communities. *S. aureus* was relatively more abundant while *S. epidermidis* and *Propionibacterium acnes* were diminished. Newborn mice colonized with *S. aureus* demonstrated strong Th17 polarization probably due to cutaneous inflammation [51]. Psoriasis arises as a result of the interaction between hyperproliferative keratinocytes and infiltrating immune cells. Immune responses on the skin are regulated by commensal microbiota that reside on the skin surface. For instance, *S.epidermis* colonization shapes the skin’s T cell network, bolstering cutaneous CD8+ T cells to produce interferon gamma, (IFNγ) and interleukin-17A (IL-17) effector functions which protect against pathogenic bacteria, *Leishmania* and *C. albicans* and also prevents exposure to *S.aureus,* hindering inflammatory diseases, which are strongly associated with psoriasis [54]. *Corynebacterium accolens*, in turn protects against *S. epidermidis* and *C. albicans* through the expansion of IL-17-producing dermal γδ T cells thus maintaining cutaneous homeostasis [54]. Disruption of this homeostasis, resulting in *S.aureus* abundance and *S.epidermis* attenuation is characteristic of psoriasis [51]. Interestingly, psoriatic patients tend to develop metabolic syndromes such as cardiometabolic diseases, abdominal obesity, diabetes mellitus and dyslipidemia. Researchers have identified common inflammatory processes, including genetic and immune responses that drive the pathology of psoriasis, cardiovascular comorbidities and immunological diseases [71]. Nevertheless, there is strong evidence that rectifying microbial composition can have a therapeutic effect, in the improvement of psoriasis and associated diseases. For example, fecal transplant to a 36-year-old male patient diagnosed with severe psoriasis for 10 years and inflammatory bowel syndrome for 15 years improved the two conditions significantly [72].

Likewise, oral intake of *Lactobacillus pentosus* GMNL-77 significantly decreased erythematous scaling lesions in imiquimod-treated mice with epidermal hyperplasia and psoriasis-like skin inflammation, with decreased tumor necrosis factor-alpha (TNF-α), interleukin (IL)-6, and IL-23/IL-17A axis-associated cytokine (IL-23, IL-17A/F and IL-22) levels in the skin and reduced IL-17- and IL-22-producing CD4+ T cells in the spleen. Additionally, ethanol extract (SEL001), isolated from *Lactobacillus sakei* proBio-65, exhibited protective effects on imiquimod-treated psoriasis-like skin inflammation in a mouse model, with decreased gene expression levels of IL-19, IL-17A and IL-23 [71].

Psoriasis was most common in CAs (3.6%), followed by AAs (1.9%), and LAs (1.6%) [51]. Though population-based studies on the prevalence of psoriasis have not been conducted in Africa, clinic-based studies have found differing prevalence rates dependent upon location, with higher prevalence (2.6–3.3%) in eastern African countries (Kenya, Uganda and Tanzania), compared to western African countries (0.05–0.3% in Nigeria, Mali, Angola) [52]. Risk factors may be extrinsic or intrinsic including stress, obesity diabetes mellitus, smoking, air pollution, arthritis, inflammatory bowel disease, alcohol, drugs, cardiovascular disease, infection, sun exposure and hypertension. Depending on genetic disposition, the aforementioned factors may trigger or exacerbate psoriasis [53,54].

### 2.7. Neurological Illness: Alzheimer’s, Parkinson’s and Multiple Sclerosis

Nasal microbiota dysbiosis has also been implicated in neurological diseases such as Alzheimer’s Disease (AZ), Parkinson’s disease (PD) and Multiple Sclerosis (MS). Some suggested mechanisms include transport of bacteria and their products from the nose to the brain, alteration of systemic or CNS-specific immunity, and reactivation of non-degradable spores from *Actinobacteria*, escorted by α-synuclein via retrograde axonal transport to hibernate in the associated cerebral nuclei [42,43]. Ethnically, there were differences in the total tremor score (F = 3.68, *p* = 0.03) found among subjects with Essential Tremors (ET). The CA group had a mean total tremor score that was 6.1 points lower than that of the Hispanic group (*p* = 0.07) and 7.2 points lower than that of the AA group (*p* = 0.05) [46]. These phenotypic differences may be reflective of genotypic differences or differences in exposure to environmental factors that influence tremor as well as variability in microbiota composition. Risk Factors for ET include exposure to neurotoxic compounds such as *β*-carboline alkaloids and ethanol, exposure to pesticide, lead, and tobacco [47,48] Multiple members of the microbiota such as *Escherichia*, *Lactobaccillus*, *Bifidobacterium*, *Enterococcus* and *Truchuris* produce neurotransmitters and neuropeptides such as dopamine, acetylcholine, gamma-aminobutyric acid, serotonin (5-hydroxytryptamine) and brain-derived neurotrophic factor [73]. These metabolites are known to be vital for brain development and functioning. A bidirectional relationship between the body microbiota and the brain suggests that microbiota-dependent signals can stimulate the nervous system and there may be a link between neuronal activation and T_reg_ cell differentiation. This may explain the immunoregulatory impacts of the microbiome. In a mouse study, disruption or absence of the microbiota impaired the function of the blood–brain barrier, altered cortical myelination and hippocampal neurogenesis, decreased cognitive function and memory formation, and decreased social and anxiety-like behavior. Microbiota-derived short-chain fatty acids have also been shown to promote the differentiation and function of microglia and macrophages in the brain [74]. They also play a significant role in the appearance of motor deficits mediated by the neuronal protein α-synuclein in a mouse model of Parkinson’s disease [75]. Risk factors for Parkinson’s Disease include head injury, family history of Parkinson’s disease, depression, and trauma [44]. In addition, patients diagnosed with obstructive sleep apnea (OSA) had associated nasal microbiome diversity correlated with the severity of the disease. *Streptococcus*, *Prevotella* and *Veillonella* were more prevalent in severe OSA cases and despite treatment, the composition of the nasal microbiota remained unchanged [40]. OSA has been suggested to accelerate the onset of mild cognitive impairment and AZ and could be an independent risk factor PD. In the early stages of AZ, continuous positive airway pressure (CPAP) treatment may slow down disease progression, thus OSA screening can be a timely intervention in these patients [76]. Risk factors of OSA include obesity, rhinitis, and adenoid hypertrophy [41] while diet, the immune system, mitochondrial function, metal exposure, and infection impacts the risk of AZ occurring [45].

## 3. Antibiotic Properties of Nasal Microbiome and Impacts on Local Immune Response

### 3.1. Antibiotic Microorganisms

Human nasal microbiota is known to compete for resources by production of antibiotics. Consequently, probiotics containing bacteria producing antimicrobial peptides such as bacteriocins are being developed to maintain or restore a host’s natural microbiome. Zipperer et al., report that commensal nasal human-associated bacteria, *Staphylococcus lugdunensis*, produce lugdunin, a cyclic bioactive thiazolidine-containing peptide, which serves as an antibiotic and causes a significant reduction in *S.aureus* carriage rate in humans. Lugdunin was found to be disease resistant and bactericidal to major pathogens in animal models and particularly, prevents colonization by *S. aureus*, which is known to cause invasive infections and life-threatening conditions [77]. In addition, the mucosal lining of the upper airways houses several immune cells, as part of the innate immune system to combat the entry of invading microbes. When a mucosal barrier is disrupted, such as a defect in ciliary function, mucociliary clearance, etc., bacterial colonization, bacterial invasion and further barrier disruption may occur [78]. In such cases, antibiotic nasal microbiome may help prevent disease. For example, *Corynebacterium propinquum* produces an iron-chelating siderophore that corresponds with its ability to inhibit coagulase-negative staphylococci and compete for limited iron in the human nasal cavity [79]. Additionally, *Staphylococcus epidermidis*, has been shown to produce antimicrobial peptides in the nasal epithelium which efficiently reduce pathogen colonization [80].

### 3.2. Impact of Nasal Microbiome on the Immune System

The microbiome and the immune system are closely connected. Nasal microbiota has also been shown to play critical roles in mucus production, development of the mucosa-associated lymphoid tissue, regulation of IgA production, tissue remodeling processes, paracellular transport across tight junctions, neuroinflammatory responses and the onset and progression of allergic or nonallergic inflammation [81]. *Lactobacillus* genus strains, for instance, have been suggested in creating an effective barrier against the infiltration of the coronavirus especially within populations that suffered less severe consequences despite noncompliance with health recommendations. *Lactobacilli*, a lactic acid producing bacteria, may thus be useful in boosting a host’s immune system and in preventing spread, in some populations compared to others [82]. Another example is the human nasal commensal *S. epidermidis* which modulates IFN-λ-dependent innate immune mechanisms in the nasal mucosa and mediates human antiviral responses, preventing the spread of IAV and suppressing IAV replication in the nasal mucosa [80]. Influenza was also shown to alter the mouse nasal microbiome via a type III IFN-STAT1-dependent mechanism and expand the nasal microbiome, increasing the colonization and aspiration of *S. aureus* and *S. pneumoniae* into the airway from the nasopharynx and restructuring the abundance of specific genera, such as *Klebsiella* and *Aerococcus*. These changes have been linked to the IFN cascade and are correlated with increased type I IFN levels. Both type I and III IFN signaling were similarly found to hinder clearance of *S. aureus* from the nasopharynx in the context of superinfection [36]. Type I IFNs are involved in the formation and activation of the inflammasome, the subsequent activation of caspase-1 and generation of the proinflammatory cytokines interleukin 1β (IL-1β) and interleukin 18 [83]. Dysregulated inflammasome activity is a key mediator of infections and airway inflammation in lung diseases and asthma. NLRP3 inflammasome that the inflammasome may also play a role in shaping the composition of the microbiota and in maintaining hemostasis. The absence of the NLRP6 inflammasome leads to the acquisition or expansion of potentially pathogenic members of the microbiota [84]. These interactions between the nasal microbiome and the immune system are still yet to be fully understood. Studies show that pathogenic bacteria in allergic rhinitis (AR) patients are suppressed and remained suppressed following immunotherapy (IT) and that AR occurs as part of a Th2-predominant immune pathway and IT effectively attenuates the IgE response and adjusts the immune response toward regulatory T cells and cellular immunity [85]. Similarly, treatment with non-glucocorticoid immunosuppressive therapy was associated with ‘healthy’ nasal microbiota in patients with GPA while GPA patients who were off immunosuppressive therapy had more dysbiosis (weighted UniFrac *p* = 0.01) [30]. Nasal carriage of *S. aureus* leads to an upregulation of the expression of the pattern recognition receptor, Toll-like receptor 2 (TLR2), and the expression of two principal epithelial antimicrobial peptides, human β-defensin-2 and human β-defensin-3 [30]. Commensal microbiota (*Legionella pneumophila*) also regulates the generation of virus-specific CD4 and CD8 T cells and antibody responses following respiratory IAV infection and provides signals leading to the expression of mRNA for pro–IL-1β and pro–IL-18 at steady state [86].

## 4. Diet, Behavior and Environmental Changes and Impacts on Respiratory Disease

The epidemiological triad highlights the influence of the agent, environment and host in disease onset [1]. Dynamic environmental exposures such as chemicals and tobacco use can alter health status. Social factors such as violence, poverty, poor access to health care, and limited access to health insurance can create a structure in which health disparities and health inequities can flourish. The microbiome plays an important role in determining individual and population health. The immune system for instance is one way the microbiome integrates the social and physical environment in disease outcomes. Understanding the synergistic detrimental effects adverse environmental changes can have on the immune system, the growth of pathogenic microbiota and health can provide more insights into mitigating health disparities [2]. Take, for instance, poverty, which has a direct impact on asthma due to the exposure of lower income individuals to environmental and lifestyle risk factors. Minority children with asthma suffer a disproportionate burden of asthma morbidity. Although National asthma guidelines recommend the use of environmental control practices as part of a comprehensive approach to asthma management, Black and Hispanic children with asthma were less likely to use mattress covers and pillow covers compared to white children in these four states. Smoking avoidance was less likely among black children but more likely among Hispanic children compared to white children. Both black and Hispanic children were more likely to live in a home without pets and without carpets compared to white children [87].

### 4.1. Diet

Dietary changes, including increases in high-fat and low fiber diets have been implicated in the global increase of airway allergic diseases (AAD) such as asthma and allergic rhinitis. According to the ‘microflora hypothesis’, long-term deficiency of fiber intake causes significant perturbations in microbial community composition and undermines normal mucosal immune tolerance, leading to an increased risk of developing allergic airway diseases or other inflammatory diseases [88] Data obtained from a mouse study, using control and AAD model mice fed with 4% standard-fiber chow, and a 1.75% low-fiber chow and sensitized and challenged with ovalbumin (OVA) to induce airway allergic inflammation showed that high dietary fiber intake increased the proportion of *Bacteroidetes* and *Actinobacteria*, and decreased *Firmicutes* and *Proteobacteria* in the intestines. Levels of probiotic bacteria, such as Lactobacillus and Bifidobacterium, were also significantly heightened and the balance of Th1/Th2 immunity was increased leading to suppressed allergic inflammatory responses [89].

### 4.2. Tobacco/Smoking

Perturbations in nasal microbiota are also frequently seen in smokers who tend to have the increased colonization of pathogenic bacteria such as *Fusobacterium*, *Gemella*, and *Neisseria* and in the genera *Staphylococcus*, *Streptococcus*, *Corynebacterium* and *Propionibacterium* [90,91]. Research findings also suggest that dysbiosis in nasal microbiota may be a consequence of increased pneumococcal colonization owing to cigarette smoke exposure [90]. Likewise, passive exposure to tobacco smoke was significantly linked to elevated colonisation of pathogenic bacteria and polymicrobial growth of pathogenic bacteria such as *Staphylococcus aureus*, *Haemophilus influenzae* and *Moraxella catarrhalis*, were more often colonised in the middle nasal meatus in children exposed to SHS (*p* = 0.031, *p* = 0.001, *p* = 0.05, respectively) in middle nasal meatus compared to non-exposed children [91]. In addition, IgA and IgG, were detected at higher concentrations in children exposed to tobacco smoke (*p* = 0.047, *p* = 0.031, respectively). In the e-cigarette users, there was a significant decrease in the relative abundance of bacteria in the genera *Propionibacterium*, *Moraxella* and *Shuttleworthia* [92].

### 4.3. Environment

Variations within the environment have been linked to alterations in bacterial diversity in the airway over time [93]. For example, pollution in industrial cities has been shown to cause an increase in the population of coagulase positive *Staphylococci*, enhance adhesion activity of *Staphylococci*, and bolster destructive processes in the epithelium [94]. In dairy farmers, compared to non-dairy farmers, increased microbial diversity owing to occupational exposures resulted in a lower burden of *Staphylococci*, independent of the abundance of *Corynebacterium* [95]. Communities living in proximity and exposed to the same environmental conditions share significant similarities in bacterial diversity and composition. Relative abundance of microbiota differs as environmental conditions diverge. The microbial distribution in the nasal sinuses of older people living in nursing homes was compared to adults within similar age ranges living in the surrounding community and similar bacterial composition was found with altered abundances among nursing home dwellers [96]. Similarly, there are marked differences in nasal microbiota between rural communities and industrial or urban cities. The nasal microbiota in a rural sample from Egypt had higher and distinct diversity with a significant increase in the representation of *Actinobacteria* and *Bacteroidetes* (*p* = 0.004, *p* = 0.01, respectively) and a predominance of Corynebacterium, *Staphylococcus*, *Alloiococcus*, and *Peptoniphilus*. In contrast, the industrial group showed a significant increase in relative abundance of phylum *Proteobacteria* (*p* = 0.02) and was dominated by *Staphylococcus*, *Sphingomonas*, and *Moraxella* [97]. Even in hospitalized settings, the ventilation conditions of the environment have been suggested to impact the proportion of shared nasal microbial communities. Compared with the non-hospitalized group, the hospitalized group had a higher proportion of surface microbiota in their nasal samples, which led to a higher risk of human-related microorganisms or pathogens colonizing the nasal cavity [98].

## 5. More on Health Disparities and Limitations

Geographical heterogeneity in the prevalence of respiratory illness has also been reported worldwide (Table 2). For example, the prevalence of adult-onset asthma was shown to be disparate across different ethnic groups. Turkish, Moroccan and South-Asian Surinamese groups (4.9–6.0%) had a higher adjusted prevalence of adult-onset asthma compared to the Dutch, Ghanaian and African Surinamese origin groups (2.4–2.6%) in a study utilizing cross-sectional data of 23,356 participants [99]. Similarly, significant disparities in allergy prevalence and microbiota were recorded between the young people in Finnish and Russian Karelia. Microbial diversity and an abundance of *Actinobacter* were significantly higher in Russian populations compared to Finnish. Comparatively, asthma, hay fever, atopic eczema, self-reported rhinitis, and atopic sensitization, were 3- to 10-fold more common in Finland [100]. There is limited data available showing the various microbiome populations of ethnic populations within the United States. For example, analysis of data retrieved from the National Cancer Institute’s Surveillance, Epidemiology and End Results Database, 1988–2010, show that sinonasal carcinomas showed poorer disease-specific survival for black versus white patients and significantly poorer disease-specific survival for sinonasal squamous cell carcinoma was also found for both black (*p* = 0.01) and Hispanic (*p* = 0.01) patients compared with white patients. In both cases, when controlling for tumor characteristics, the disease-specific survival disparity was eliminated for black and Hispanic patients signifying that ethnic populations presented with more advanced disease [101]. However, there was no data collected to account for variations in nasal microbiome and the impact on the progression and severity of the disease. Similarly, the incidence of paranasal sinus squamous cell carcinomas (PNSSCC) varied among different ethnic groups. Compared with whites, the incidence was higher among African Americans (RR 1. 63; 95% CI, 1.39, 1.90) and among all other racial groups (RR, 1.78; 95% CI: 1.53–2.07). After adjusting for age, sex, disease stage, tumor site, and treatment, mortality among AA patients also was increased (HR, 1.22; 95% CI, 1.04–1.43) [102]. In pregnant women, AA was shown to have more prior diagnosis of asthma and was more sensitized with high IgE levels, after controlling for socio-economic or environmental variables, when compared to CA counterparts [103]. Furthermore, analysis of pediatric patients’ populations receiving Endoscopic Sinus Surgery (ESS) demonstrated that AA and LA children disproportionately underwent more urgent and emergent ESS, and more advanced presentation [104]. The disparities in the incidence, prevalence and mortality among the groups need to be analyzed with regard to the population of nasal microbiota. Uncovering the role of the microbiome in health disparities could enhance our understanding of disease risks, shedding light on how social and environmental exposures interact with biology and guide efforts to reduce and eliminate health disparities.

Another limitation is that causes of health disparities are multifarious. Other studies have reported higher prevalence rates of allergic conditions such as asthma in long term migrants to a Western country compared with recent immigrants. For instance, long term Chinese immigrants to Australia had more bacterial carriage than short-term immigrants, 29 bacteria (30.2%) vs. 16 bacteria (16.7%), respectively and a significantly increased prevalence of *S. aureus* and *S. pneumoniae* (14/56, 25%; *p* = 0.012 and 43/56, 76.8%; *p* = 0.002, respectively) [105]. These findings suggest an important influence from environmental factors on the development of asthma such as immunity from early life exposures to infectious agents in the birth country.

Finally, previous studies have shown that crosstalk exists between microbiome and epigenome and that Host-microbial interactions may be involved in pathophysiology of diseases [107]. A single nucleotide polymorphism (SNP) significantly associated with DNA methylation of a CpG site (cg25024579) at the *FLJ22447* locus was found in Puerto Rican children and adolescents. This SNP was further associated with increased expression of *KCNJ2-AS1* in nasal airway epithelium causing severe asthma exacerbations in Latino youth. Changes in the expression of *KCNJ2-AS1* were also implicated in atopic asthma in Puerto Rican children and adolescents [108].

These aforementioned factors and many more not discussed in this paper may contribute to the development of health disparities and more research needs to be carried out to properly categorize their influences on each other.

## 6. Potential Pitfalls

Respiratory and neurological Illnesses are heterogeneous diseases that have differences among various ethnic and racial groups. Thus, there is a growing need for the development of future, targeted treatments and for personalized medicine approaches. This discussion investigates three unique but overlapping areas of health research—the microbiome, biological processes, and social and physical environmental factors. Variation in disease phenotype is multifactorial, including differences in access to health care, immunity, environment, and the host microbiome. The discussion opens a wide range of possibilities for the individualized treatment of utilizing the knowledge of microbial signatures and their interactions with the immune system. By bringing more awareness to the fact that the presence of distinct microbial opportunities may present an opportunity for designing treatment technologies that help mitigate disease and decrease the staggering gaps in health status among various ethnic groups.

It is important to note that this is not an all-encompassing study and care must be taken to not generalize as within each ethnic population, outliers are bound to exist and in the profession of medicine, the focus remains to do no harm and to treat each patient as though they were solely in the population. More research needs to be undertaken to increase the pool of data that is currently available so that more studies can be carried out to fully comprehend associations and correlations among the multiple factors discussed above. However, knowledge of predispositions can allow for more attention and care to be provided for those that may be disproportionately affected and to provide a more robust form of assessing disease. For instance, ethnic minorities in the United Kingdom versus ethnic minorities in the United States may present with varying microbiome signatures although they may be classified under a similar racial affiliation as both groups are exposed to different social constructs, environmental factors, access to healthcare, food, etc. Nevertheless, understanding that those varying factors play a role in the pathogenesis of disease, clinicians can provide more encompassing care. 

## 7. Concluding Remarks

The nose is an important site of pathogen colonization and the indigenous microbiota of the nasal cavity plays important roles in human health and disease. Nasal microorganisms modulate immune function and thus are likely to be potent contributors to certain health disparity diseases and conditions. Due to the complex and multifactorial etiology of modern diseases, understanding the triggers responsible for shifts in microbial communities across many disease states and the impact that these shifts have on the host immune system, and genetic variation, can provide a better context to comprehend and decrease disease burdens in various ethnic groups. Given the recent COVID-19 pandemic outbreak and the increased mortality reported among ethnic minorities in the United States, increased study of the role of nasal microbiota may offer new insights into numerous adaptable therapeutic strategies for the treatment of respiratory illness, especially as societies begin to host increasingly multicultural populations. The COVID-19 pandemic has allowed millions of people to get accustomed to nasal swabs, thereby creating an opportunity for examination of the nasal microbiome of people from different backgrounds, race, and socioeconomic status. Furthermore, as lower airway diseases are detrimental to overall health and well-being and obtaining uncontaminated swabs from the lung remains difficult, understanding the nasal microbiome is vital to elucidate the mechanisms of lower respiratory diseases as changes in the lower airway are often reflected in changes in the nasal cavity. In addition, there is not much known about the impact of nasal microbiota on DNA modification and subsequent disease pathogenesis. Studying the relationship between nasal microbiota and epigenetic modifications is a potentially useful strategy to elucidate new ways to combat diseases and reduce disease burdens in affected populations. Grouping people by nasal microbiome populations may thus prove even more useful to identify potential risk factors that affect respiratory health than using socioeconomic status, lifestyle, or race. In addition, it may provide further insight into additional environmental factors such as pollutants that highly correlate to pathogenic microbial variants. Since people with lower socioeconomic status tend to live in highly polluted areas, they may present with greater risk of respiratory illnesses. Further studies involving microbial interactions within the host system are needed to elucidate potential therapeutic interventions.

## Figures and Tables

**Table 1:** Disparities in the abundance of microbiota are linked to disease and may vary across ethnic groups.

Disease	Greater Abundance Linked to Disease	Affected Group	Known Risk Factors
Chronic otitis media with effusion (COME)	*Corynebacterium*, *Streptococcus*, *Moraxella* [16]	Caucasian Americans (CAs) [17]	allergy or atopy, upper respiratory tract infection, snoring, acute otitis media, passive smoke exposure and low social status [17,18]
Pediatric Asthma	*Corynebacterium* [19]	Puerto-Ricans (PRs), African Americans (AAs) [20]	parental asthma, prenatal environmental tobacco smoke, having cats and prematurity [21,22]
Chronic Rhinosinusitis (CRS)	*Corynebacterium*, *Burkholderia* [23,24]	Latino Americans (LAs) [24]	Asthma, genetics, GERD, rheumatoid arthritis, migraine, cigarette smoking [25]
COVID-19	*Salmonella*, *Scardovia*, *Serratia* and *Pectobacteriaceae* [26,27]	AAs and Asian Americans (AS) [28]	Hypertension, coronary artery disease, history of stroke, diabetes, obesity, severe obesity, chronic kidney disease, asthma, and chronic obstructive pulmonary disease [29]
Granulomatosis with Polyangiitis (GPA)	*Staphylococcus aureus* [30]	Not Distinguishable [31]	Animal (horses) exposure, history of bronchiectasis, autoimmune disease, chronic renal impairment, Pulmonary fibrosis [32,33]
Acute Otitis Media (AOM)	*Haemophilus*, *Moraxella*, and *Neisseria* [16]	CAs [34]	Out-of-home daycare, multiple children living in the home, and mother’s multiparity [34]
Influenza B virus (IBV)	*Streptococcus* species and *Prevotella salivae* [35]		Diabetes, chronic respiratory disease [36]
Influenza A virus (IAV)	*Staphylococcus aureus*, *Staphylococcu pneumoniae*, *Klebsiella* and *Aerococcus* [37]		
Acute Respiratory Distress Syndrome (ARDS)		CAs [38]	abuse of alcohol and tobacco, malnutrition and obesity [39]
Obstructive Sleep Apnea (OSA)	*Streptococcus*, *Prevotella* and *Veillonella* [40]		Obesity, rhinitis, adenoid hypertrophy [41]
Parkinson’s Disease (PD)	*Actinobacter* [42,43]		OSA, Head injury, family history of trauma and depression, family history of PD [41,44]
Alzheimer’s Disease (AZ)			OSA, diet, the immune system, mitochondrial function, metal exposure, and infection [41,45]
Essential Tremors (ET)		AAs, LAs [46]	Exposure to neurotoxic compounds such as *β*-carboline alkaloids and ethanol. Exposure to pesticide and lead. Tobacco exposure [47,48]
Atopic Dermatitis (AD)		AAs [49]	Viral and bacterial skin infections, neuropsychiatric diseases, family history, smoking, allergy, maternal asthma, and dogs [50]
Psoriasis		CAs, Eastern African [51,52]	Stress, diabetes mellitus, obesity, smoking, air pollution arthritis, inflammatory bowel disease, alcohol, drugs, cardiovascular disease, infection, sun exposure, hypertension [53,54]

**Table 2:** Differences in health outcomes are based on interaction with microbiome variation and other factors.

Disease	Varying Factor	Higher Prevalence	Lower Prevalence
Adult- Onset Asthma	Citizenship	Turkish, Moroccan and South-Asian Surinamese [99]	Dutch, Ghanaian and African Surinamese origin groups [99]
Allergies (asthma, hay fever, atopic eczema, self-reported rhinitis and atopic sensitization,)	Citizenship	Finland [100]	Russia (High *Actinobacter)* [100]
sinonasal carcinomas & paranasal sinus squamous cell carcinomas	Ethnicity	AAs [101,102]	CAs [101,102]
Endoscopic Sinus Surgery	Ethnicity, Stage at presentation	AAs, LAs [104]	Cas [104]
Allergies	Amount of Exposure to Environment	Long Term Western Migrants (*S. aureus* and *S. pneumoniae)* [105]	Recent Western Migrants [105]
Asthma	Ethnicity, Genetics	PR LAs [106]	

## Data Availability

Not Applicable.
